# Human Papillomavirus Type 18 E6 and E7 Genes Integrate into Human Hepatoma Derived Cell Line Hep G2

**DOI:** 10.1371/journal.pone.0037964

**Published:** 2012-05-24

**Authors:** Tianzhong Ma, Zhongjing Su, Ling Chen, Shuyan Liu, Ningxia Zhu, Lifeng Wen, Yan Yuan, Leili Lv, Xiancai Chen, Jianmin Huang, Haibin Chen

**Affiliations:** 1 Department of Histology and Embryology, Shantou University Medical College, Guangdong Province, China; 2 Department of Biochemistry and Molecular Biology, Shantou University Medical College, Guangdong Province, China; 3 Pediatric Endocrine Unit, Massachusetts General Hospital for Children and Harvard Medical School, Boston, Massachusetts, United States of America; Health Canada, Canada

## Abstract

**Background and Objectives:**

Human papillomaviruses have been linked causally to some human cancers such as cervical carcinoma, but there is very little research addressing the effect of HPV infection on human liver cells. We chose the human hepatoma derived cell line Hep G2 to investigate whether HPV gene integration took place in liver cells as well.

**Methods:**

We applied PCR to detect the possible integration of HPV genes in Hep G2 cells. We also investigated the expression of the integrated E6 and E7 genes by using RT-PCR and Western blotting. Then, we silenced E6 and E7 expression and checked the cell proliferation and apoptosis in Hep G2 cells. Furthermore, we analyzed the potential genes involved in cell cycle and apoptosis regulatory pathways. Finally, we used in situ hybridization to detect HPV 16/18 in hepatocellular carcinoma samples.

**Results:**

Hep G2 cell line contains integrated HPV 18 DNA, leading to the expression of the E6 and E7 oncogenic proteins. Knockdown of the E7 and E6 genes expression reduced cell proliferation, caused the cell cycle arrest at the S phase, and increased apoptosis. The human cell cycle and apoptosis real-time PCR arrays analysis demonstrated E6 and E7-mediated regulation of some genes such as Cyclin H, UBA1, E2F4, p53, p107, FASLG, NOL3 and CASP14. HPV16/18 was found in only 9% (9/100) of patients with hepatocellular carcinoma.

**Conclusion:**

Our investigations showed that HPV 18 E6 and E7 genes can be integrated into the Hep G2, and we observed a low prevalence of HPV 16/18 in hepatocellular carcinoma samples. However, the precise risk of HPV as causative agent of hepatocellular carcinoma needs further study.

## Introduction

Epidemiological studies have shown that HPV infection is the main etiological factor for cervical cancer [1] and high-risk HPV type viral DNAs are frequently integrated into the host cell genome in HPV-related cervical carcinomas [2]. This integration has been associated with dysregulation of E6 and E7 viral genes expression, which accounts for the major oncogenic activity of the HPV DNA [3]. Expression of these genes can lead to immortalization of keratinocytes, the natural host cells of HPV [4]. However, little information is available about the integration of HPV into human liver cells.

The human hepatoma derived cell line Hep G2 was derived from biopsies taken during extended lobectomy of a 15-year-old Caucasian male from Argentina [5]. This cell line has been used in many laboratories around the world and we discovered that the Hep G2 cell line contained the integrated DNA of HPV 18. All the HPV viruses live exclusively in the superficial tissues that cover our body parts: the skin, the lining of the genital organs, urethra, bladder, rectum, vocal cords, and esophagus. It remains unclear whether there is an association between HPV infection and hepatocellular carcinoma. Our interest in such a putative association was the impetus that led us to investigate the expression of E6 and E7 oncogenes in the Hep G2 cell line, and furthermore, to see if such expression is required for the maintenance of the proliferative and malignant phenotypes of Hep G2 cell line.

## Results

### Immunohistochemistry revealed that the Hep G2 cell line was typical of liver cells

**Figure 1:**
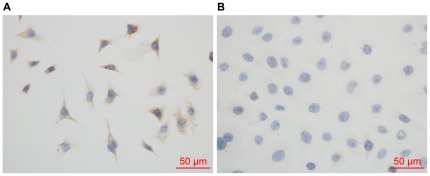
Immunohistochemistry demonstrated that the Hep G2 cell line was of characteristics typical of liver cells. Immunohistochemical evaluation of Hep G2 cells with anti-human hepatocyte antibody indicated that the Hep G2 cell cytoplasm (A), but not HeLa cells (B), exhibited typical hepatocyte antigens.

In order to characterize the Hep G2 cell line more explicitly, we used the anti-human hepatocyte antibody to verify the characteristics of the hepatoma cells. Anti-human hepatocyte immunohistochemical evaluation of Hep G2 with the hepatocyte-specific marker confirmed that the Hep G2 cell line was positive in liver cell antigens (Fig. 1A), but the HeLa cells were negative (Fig. 1B).

### Hep G2 cells with integrated HPV 18 DNA expressed E6 and E7 mRNAs and proteins

**Figure 2:**
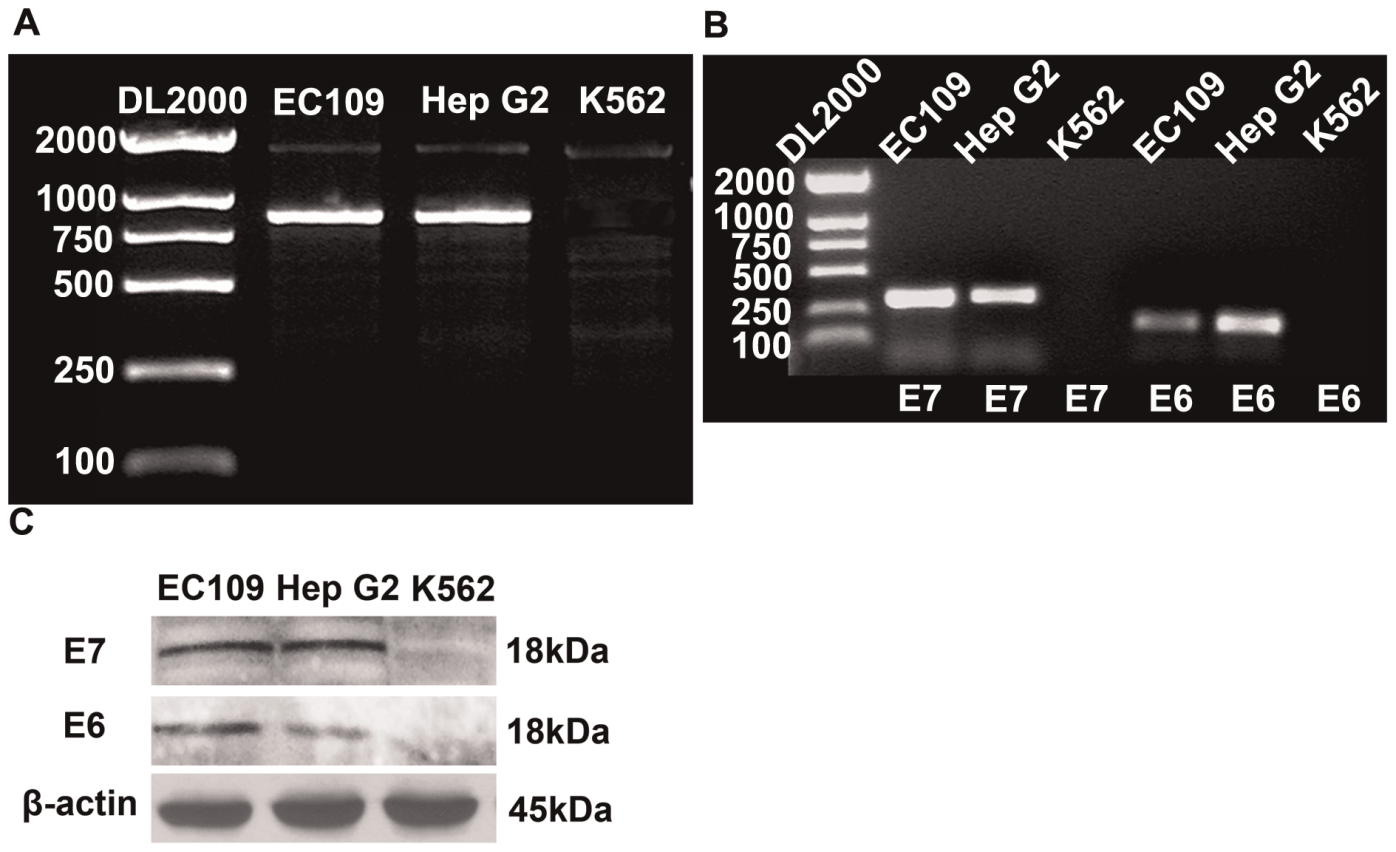
Hep G2 cells with integrated HPV 18 DNA expressed E6 and E7 mRNAs and proteins. (A) PCR amplification of HPV 18 E6E7 gene was assessed in samples from Hep G2, EC109, and K562 cells. Then an amplified fragment of 847 bp was present in both Hep G2 and EC109 cells (HPV 18 positive), but absent in K562 cells (HPV 18 negative). (B) The expression of HPV 18 E6 and E7 mRNA was detected by RT-PCR. The expected fragments of E6 (196 bp) and E7 (332 bp) were present in both Hep G2 and EC109 cells, but not in K562 cells. (C) Western blotting showed the expression of the HPV 18 E6 and E7 proteins. EC109 and K562 were used as controls. β-actin was used as an internal control.

An amplified fragment of 847 bp was present in both the Hep G2 and EC109 cells (HPV 18 positive) while it was absent in K562 cells (HPV 18 negative), as was expected (Fig. 2A).

Transcription of the HPV 18 E6 and E7 genes in the Hep G2 and EC109 cell lines were evaluated by RT-PCR. The results showed that the expected fragments of E6 (196 bp) and E7 (332 bp) were present in both Hep G2 and EC109 cells, but not in K562 cells (Fig. 2B). Western blot analysis of cell extracts was also carried out to determine whether mRNA expression was correlated with translation of the gene products. Again, a specific protein band (18 kD) of E6 and E7 was observed in both Hep G2 and EC109 cells, but not in K562 cells (Fig. 2C). β-actin was used as an internal control.

### Inhibition of both E6 and E7 genes expression by HPV 18 E7 siRNA

**Figure 3:**
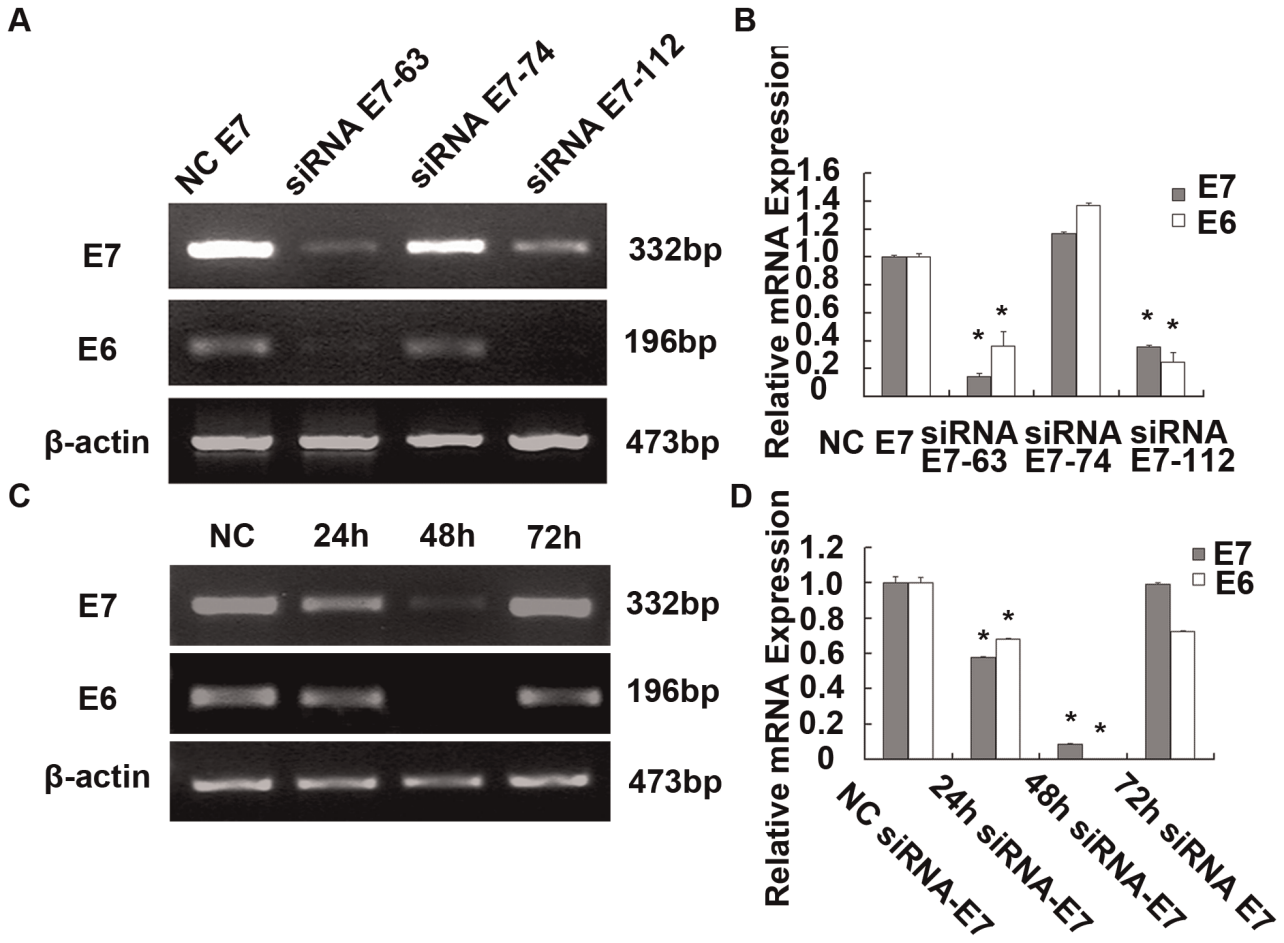
HPV18 E6 and E7 mRNA and relative protein expressions in Hep G2 transfected with E7-siRNA. (A) Effective siRNAs were screened using RT-PCR assays from three siRNAs targeting the E7 gene and two of three siRNAs (siRNA E7-63 and siRNA E7-63 112) were more effective. (B) The level of E7 mRNA in Hep G2 cells transfected with siRNA E7-63 and siRNA E7-112 decreased to 14.51%±2.06% and 35.89%±1.00% respectively compared with that of the NC siRNA E7 (Negative control) transfected cells (p<0.01). (C) The expression of E7 and E6 mRNA was assessed through RT-PCR at different time after transfection. (D) At 24 h and 48 h after transfection, the level of E7 mRNA in Hep G2 cells transfected with siRNA E7 decreased to 57.93%±0.31% and 8.79%±0.42% respectively compared with that of the NC siRNA E7 transfected cells (p<0.01).

We designed three siRNAs targeting E7 gene and screened for more effective siRNAs using RT-PCR assay. We found that two of three siRNAs (siRNA E7-63 and siRNA E7-112) were more effective (Fig. 3A). The level of E7 mRNA in Hep G2 cells transfected with siRNA E7-63 and E7-112 decreased to 14.51%±2.06% and 35.89%±1.00% respectively compared with that of the NC E7 (negative control) transfected cells. The level of E6 mRNA in Hep G2 cells transfected with siRNA E7-63 and E7-112 decreased to 36.50%±10.12% and 24.50%±7.32% respectively compared with that of the NC E7 (negative control) transfected cells (p<0.01, Fig. 3B). To overcome limitations of RNAi experiments, such as off­target effects, we mixed siRNA E7-63 and E7-112 to obtain our siRNA pools. The expression of E7 and E6 at mRNA and protein levels was assessed at different times after transfection. There were significant decreases in E6 and E7 mRNA levels at 24 and 48 hours after siRNA treatment when compared with that of control siRNA treated cells (Fig. 3C). At 24 h and 48 h after transfections, the level of E7 mRNA in Hep G2 cells transfected with siRNA E7 pools decreased to 57.93%±0.31% and 8.79%±0.42% respectively compared with that of the NC E7 transfected cells. At 24 h and 48 h after transfections, the level of E6 mRNA in Hep G2 cells transfected with siRNA E7 pools decreased to 68.14%±0.53% and 0.63%±0.02% respectively compared with that of the NC E7 transfected cells (p<0.01, Fig. 3D).

### Knockdown of HPV 18 E7 and E6 genes inhibited cell growth

**Figure 4:**
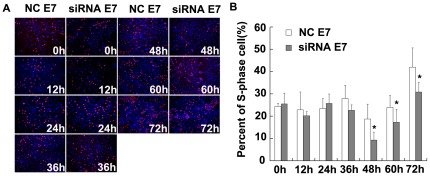
Inhibition of HPV 18 E7 gene inhibited cell growth in Hep G2. (A) Hep G2 cells transfected with E7-siRNA or control siRNA were evaluated in EdU assays at 0 h, 12 h, 24 h, 36 h, 48 h, 60 h and 72 h after transfection. After transfection of Hep G2 cells with E7-siRNA, a time-dependent reduction of cell proliferation was observed at 48 h, 60 h and 72 h. (B) At 48 h, 60 h and 72 h following E7-siRNA transfection, the percents of S-phase cells were 9.39%±3.55%, 17.29%±5.85% and 30.87%±4.26% compared with 18.74%±6.66%, 24.03%±5.35% and 41.97%±8.73% in NC-siRNA transfected cells (p<0.05).

To determine whether the inhibition of HPV 18 E7 and E6 genes was sufficient to suppress the proliferation of Hep G2 cells, cells were assessed using EdU assays for analyzing the S-phase fraction (SPF). After transfections of Hep G2 cells with E7-siRNA, a time-dependent reduction of cell S-phase fraction was observed at 48 h, 60 h and 72 h (Fig. 4A). At 48 h, 60 h and 72 h following E7-siRNA transfections, the percents of S-phase cells were 9.39%±3.55%, 17.29%±5.85% and 30.87%±4.26% compared with 18.74%±6.66%, 24.03%±5.35% and 41.97%±8.73% in NC-siRNA transfected cells (P<0.05, Fig. 4B).

### Transfection with E7-siRNA induced apoptosis

**Figure 5:**
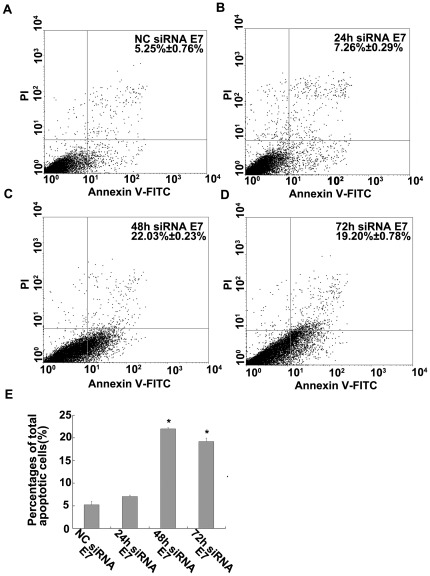
Transfection with E7-siRNA induced apoptosis. (A, B, C and D) The percent of apoptotic Hep G2 cells was measured using the Annexin V assay at 24 h, 48 h and 72 h after transfection, and then the stained cells were analyzed through flow cytometry. (E) The results showed that 7.26%±0.29%, 22.03%±0.23% and 19.20%±0.78% in siRNA E7 transfected Hep G2 cells after 24 h, 48 h and 72 h underwent total apoptosis compared with only 5.25%±0.76% in NC siRNA E7 transfected cells (p<0.05).

To assess the ratio of cell death, the Annexin V apoptosis assay was performed at 24 h, 48 h and 72 h after siRNA transfections and the stained cells were analyzed by flow cytometry in the experimental and negative control groups. As shown in Fig. 5A, B, C and D, 7.26%±0.29%, 22.03%±0.23% and 19.20%±0.78% in siRNA E7-transfected Hep G2 cells after 24 h, 48 h and 72 h underwent total apoptosis compared with only 5.25%±0.76% in NC-E7 transfected cells. The fourth and first quadrant cells were counted and expressed as the percentages of apoptotic cells. Therefore, knockdown of E7 resulted in about a 4-fold increase in apoptosis (P<0.01, Fig. 5E).

### Real-time PCR array analysis of shRNA E7 induced apoptosis and cell cycle gene transcriptional profiles

In this study, we compared the gene expression patterns of cell cycle and apoptosis signaling pathways in Hep G2 cells transfected with shRNA E7 or negative control shRNA at 48 h after transfections. A total of 40 out of 168 genes in PCR Array showed differential expressions when cells transfected with shRNA E7 and NC-shRNA were compared. Data analysis indicated that among the cell cycle PCR array, 7 genes were up-regulated and 12 genes were down-regulated after E7 gene was silenced (Table 1). Cyclin H, CDK2, E2F4, p53, Cullin3, MKI67, UBA1, p107 and others were affected. After the knockdown of E7, in Apoptosis PCR Array, 16 genes were up-regulated and 5 genes were down-regulated. Affected genes were BAK1, BCL2L10, CARD8, CASP2, CASP8, CASP14, FASLG, NOL3, CD27 and other genes (Table 2).

**Table 1:** mRNA expression of E7-related genes in Hep G2 cells after the silencing of HPV 18 E7 through RT^2^ Profiler™ Human Cell Cycle PCR Array.

Gene names	Genbank accession no.	Description	Folds up- or down-regulation
ABL1	NM_005157	C-abl oncogene 1, receptor tyrosine kinase	−2.20
ANAPC4	NM_013367	Anaphase promoting complex subunit 4	−2.13
ATR	NM_001184	Ataxia telangiectasia and Rad3 related	−2.31
CCNH	NM_001239	Cyclin H	−2.80
CDK2	NM_001798	Cyclin-dependent kinase 2	2.12
CDKN2B	NM_004936	Cyclin-dependent kinase inhibitor 2B (p15, inhibits CDK4)	2.01
CKS1B	NM_001826	CDC28 protein kinase regulatory subunit 1B	−2.09
CUL3	NM_003590	Cullin 3	−2.83
E2F4	NM_001950	E2F transcription factor 4, p107/p130-binding	3.27
MKI67	NM_002417	Antigen identified by monoclonal antibody Ki-67	−2.48
MNAT1	NM_002431	Menage a trois homolog 1, cyclin H assembly factor (Xenopus laevis)	2.04
NBN	NM_002485	Nibrin	−2.04
PCNA	NM_182649	Proliferating cell nuclear antigen	−2.08
RAD1	NM_002853	RAD1 homolog (S. pombe)	−2.29
RBL1	NM_002895	Retinoblastoma-like 1 (p107)	2.83
RPA3	NM_002947	Replication protein A3, 14 kDa	−2.08
SERTAD1	NM_013376	SERTA domain containing 1	−2.20
TP53	NM_000546	Tumor protein p53	3.20
UBA1	NM_003334	Ubiquitin-like modifier activating enzyme 1	2.44

In 84 E7-related genes, 19 genes mRNA transcript were altered markedly compared with the negative control. Standards of eligibility: folds up- or down-regulation >2.0 and *p*<0.05; n = 3.

**Table 2:** mRNA expression of E7-related genes in Hep G2 cell after the silencing of HPV 18 E7 in RT^2^ Profiler™ Human Apoptosis PCR Array.

Gene names	Genbank accession no.	Description	Folds up- or down-regulation
BAK1	NM_001188	BCL2-antagonist/killer 1	2.48
BAX	NM_004324	BCL2-associated X protein	2.07
BCL2	NM_000633	B-cell CLL/lymphoma 2	−2.30
BCL2L10	NM_020396	BCL2-like 10 (apoptosis facilitator)	−3.05
BIK	NM_001197	BCL2-interacting killer (apoptosis-inducing)	2.05
BIRC3	NM_001165	Baculoviral IAP repeat-containing 3	−2.14
CARD6	NM_032587	Caspase recruitment domain family, member 6	2.05
CARD8	NM_014959	Caspase recruitment domain family, member 8	2.13
CASP2	NM_032982	Caspase 2, apoptosis-related cysteine peptidase	2.01
CASP8	NM_001228	Caspase 8, apoptosis-related cysteine peptidase	2.03
CASP14	NM_012114	Caspase 14, apoptosis-related cysteine peptidase	3.71
CIDEA	NM_001279	Cell death-inducing DFFA-like effector a	2.23
CIDEB	NM_014430	Cell death-inducing DFFA-like effector b	2.12
FASLG	NM_000639	Fas ligand (TNF superfamily, member 6)	4.00
LTBR	NM_002342	Lymphotoxin beta receptor (TNFR superfamily, member 3)	2.04
NOL3	NM_003946	Nucleolar protein 3 (apoptosis repressor with CARD domain)	−3.92
TNFRSF10A	NM_003844	Tumor necrosis factor receptor superfamily, member 10a	−2.06
CD27	NM_001242	CD27 molecule	2.29
TRAF2	NM_021138	TNF receptor-associated factor 2	2.09
TRAF3	NM_003300	TNF receptor-associated factor 3	2.03
TRAF4	NM_004295	TNF receptor-associated factor 4	2.25

In 84 E7-related genes, 21 genes mRNA transcript were altered markedly compared with the negative control. Standards of eligibility: folds up- or down-regulation >2.0 and *p*<0.05; n = 3.

### In situ hybridization on tissue microarrays for HPV 16/18

**Figure 6:**
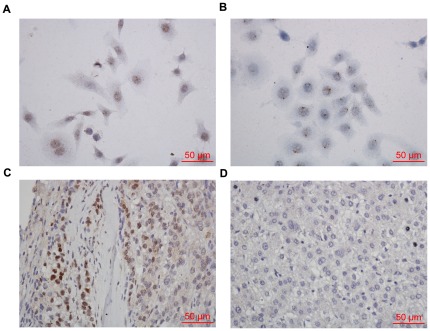
These photomicrographs show in situ hybridization results for human papillomavirus positive hepatocellular carcinoma. (A) Hep G2 cells were with the punctate signal pattern of HPV DNA. (B) HeLa cells were served as positive control. (C) Hepatocellular carcinoma was with diffuse signal pattern of HPV staining. (D) No signal was found in hepatoma carcinoma cells of this HPV- negative specimen.

The HPV signals observed in the punctate nuclear staining of Hep G2 cells (Fig. 6A) and HeLa cells (positive control) demonstrated an integrated form of HPV DNA (Fig. 6B). HPV signals in the HPV-ISH-positive case were of diffuse staining in tumor cells (Fig. 6C). No signal was found in hepatoma carcinoma cells of this HPV- negative specimen. (Fig. 6D). With regard to tumor grade, HPV16/18 was positive in 2 of 28 patients (7.1%) with grade I carcinoma, in 6 of 65 patients (8.8%) with grade II carcinoma, and in 1 of 7 patients (14.2%) with grade III carcinoma (Table 3). There was no association between carcinoma grade and HPV infection (P = 0.719).

**Table 3:** Characteristics of the Hepatocellular Carcinoma Cases With HPV Infection.

Hepatocellular carcinoma case	Subtype-specific ISH HPV 16/18	Age	Gender	Grade
1	+	61	Male	I
2	+	42	Male	II
3	+	62	Male	II
4	+	76	Male	II
5	+	45	Male	II
6	+	58	Male	II
7	+	68	Male	II
8	+	61	Male	I
9	+	60	Male	III

## Discussion

Chronic viral and bacterial infections and nematode infestations have been estimated to be associated with approximately one out of five human cancers worldwide [6]. The HPV genome has been detected in esophagus, lung, colon, ovary, breast, and prostate. However, there is little research addressing the ability of HPV to infect hepatic cells and the effect it would have upon these cells. Scinicariello F. et al detected human papillomavirus 16 and 18 in primary hepatocellular carcinoma and found that tumors in 1 of 16 samples showed HPV 16 positive and 2 of 16 samples showed HPV 18 positive [7].HPV is a simple DNA virus that has evolved to escape immune attack by a combination of stealth and interference. HPV life cycle has rare by-products to trigger immune response and E6E7 oncoproteins also target cytokine expression to modulate cell proliferation and interferon responses, contributing to immune evasion [8]. When the immune system is weakened, HPV is likely carried to the liver of the patients from a distant site via blood circulation. This may explain why HPV viremia occurs more frequently than previously believed.

Our research have shown that the hepatoma derived cell line Hep G2 was positive for HPV 18 and this infection led to the integration of viral DNA into the host genome and expression of the E6 and E7 oncogenes. For a long time, there has been controversies about the precise origin of Hep G2 cell line. The HeLa cells do contaminate a number of established cell lines. A major issue here was to determine if this line was a cervical adenocarcinoma, a carcinoma of the cervix metastatic to the liver, a primary hepatoma cell line, or a contaminated pailloma positive HeLa cell line. Anti-hepatocyte immunohistochemistry convincingly demonstrated that Hep G2 cell line was of typical characteristics of liver cells. This evidence suggested a possible association between HPV 18 infection and hepatocellular carcinoma (HCC). As a basic biological characteristic, HPV virus causes proliferation of infected host cells. This is often manifested clinically in skin or mucous membrane warts. It is unknown whether HPV plays a role in HCC carcinogenesis, or whether HPV and HBV co-infection may have a synergistic effect. Lee et al found that HPV 16 E6 oncoprotein and HBV X protein could synergistically induce the activation of the AP1 site of E element in the enhancer I (EnI) to increase the transcription activity of the HBV and other oncogenes containing an AP1 site in the promoter in human liver cell [9]. Both HBV and HPV are DNA viruses, and share, along with oncogenic retroviruses, a replication strategy involving reverse transcriptase and a characteristic life cycle that includes integration of viral DNA into the host genome [10]. Viral integration into or adjacent to the human telomerase reverse transcriptase (hTERT) gene, for example, may lead to hTERT telomerase activation, cellular immortalization, and a predisposition to carcinogenesis in liver and cervical cancers [11]. The HPV oncoproteins E6 and E7 are the primary viral factors responsible for initiation and progression of cancer. A primary target of E7 is the retinoblastoma (Rb) family of proteins that control the activity of E2F transcription factors, which are key regulators of S phase genes. Inactivation of Rb is important for the differentiation-dependent productive viral lifecycle and for tumor progression.These high-risk E6 and E7 oncoproteins then lead to cell proliferation through their association with PDZ domain proteins and, through p53 degradation, prevent the normal repair of chance mutations in the cellular genome [12]. E7 oncoprotein also alters cell cycle control through interactions with histone deacetylases, cyclins and cyclin-dependent kinase inhibitors. These factors are crucial regulators of immune evasion, cell cycle progression, telomere maintenance, apoptosis and chromosomal stability. HPV oncoproteins modify these activities to play an important role in the basic mechanisms of oncogenesis [13], [14]

In recent years, most researchers have reported that down-regulation of HPV18 E6 and E7 expression by RNAi led to retarded growth of HPV18-positive cervical cancer cell lines [15] and proposed that siRNA-induced E6 and E7 simultaneously silencing had a major therapeutic potential. When we silenced the E7 and E6 by RNAi, Hep G2 cells in the S-phase reduced and apoptosis increased. We found that silencing HPV E7 and E6 could affect the expression of genes involved in cell-cycle regulation, such as Cyclin H, CDK2, E2F4, p53, Cullin3, MKI67, UBA1, p107 and so on. In our study, the knockdown of HPV E7 could affect the genes of cell apoptosis including BAK1, BCL2L10, CARD8, CASP2, CASP8, CASP14, FASLG, NOL3 and CD27. Therefore, integration of the HPV E7 and E6 genes into Hep G2 cells may play an important role in the immortalization and carcinogenesis of this hepatoma cell line.

These findings should encourage clinicians and medical professionals to consider the effect that HPV infection has on the development, investigation, and treatment of HCC. It is standard practice to inoculate for HBV to prevent the development of chronic HBV infection and HCC. Perhaps consideration should also be given to the administration of HPV vaccine in adult immunization, or to include HPV testing as part of routine outpatient care. Patients with HCC who are HBV positive undergo liver transplantation require antiviral therapy. There is a benefit from viral suppression in the form of reducing HBV recurrence after transplantation [16]. HPV status, on the other hand, has never played a role in the consideration of liver resection versus transplantation in patients with HCC. Perhaps this stance should be investigated further.

In conclusion, our research showed that HPV 18 E6 and E7 genes can be integrated into the hepatoma cell line Hep G2, leading to the expression of the E6 and E7 oncogenic proteins. Furthermore our results demonstrated that RNAi inhibited the expression of E7 as well as E6, and down-regulation of these viral oncogenes initiated the process of growth arrest and induced apoptosis in Hep G2 cells. Although 100 tumor samples were analyzed and only 9% of cases tested positive for HPV 16/18 in hepatocellular carcinoma, further research on the carcinogenic potential of HPV infection in hepatic cells is warranted.

## Materials and Methods

### Cell lines and cell culture

The human hepatoma derived cell line Hep G2 [5] and human esophageal carcinoma cell line EC109 cell lines were obtained from the Committee of Type Culture Collection of the Chinese Academy of Sciences (Shanghai, China). The human erythroleukemia line K562 [17] and human cervical carcinoma cell line HeLa [18] were obtained from the Center for Molecular Biology of Shantou University Medical College (Shantou, China). The cell lines were maintained in Dulbecco's modified Eagle's medium (DMEM) supplemented with 10% fetal bovine serum (FBS) (Invitrogen Gibco, USA) and grown at 37°C in a humidified atmosphere with 5% CO_2_.

### Immunohistochemistry for characterizing Hep G2 cells

Cells grown on slides were fixed in paraformaldehyde for 15 min and penetrated in Phosphate buffered saline (PBS) containing 0.3% Triton X-100 for 30 min, then followed by incubation with 3% hydrogen peroxide for 25 min. Washed in PBS, slides were incubated with 1.5% normal horse serum in PBS for 30 min with the purpose of blocking nonspecific binding. After being rinsed in PBS for three times, slides were incubated overnight at 4°C with mouse anti-human hepatocyte antibody (1∶100 dilution) (Invitrogen, USA). Slides were then incubated for 30 min with biotinylated anti-mouse antibody (1∶200 dilution) (VECTOR Lab, USA) followed by 30 min in Avidin: Biotinylated Enzyme Complex at room temperature. Subsequently the antibody-antigen binding sites were identified by applying substrate diaminobenzidine. Finally, slides were dehydrated, and mounted. HeLa cells were served as controls.

### Polymerase chain reactions (PCR)

PCR amplification of HPV 18 E6 and E7 genes was performed using Pfu DNA polymerase (Generay, Shanghai, China) and the following HPV 18 E6 and E7 primers (forward 5′GACACTAGTACTATGGCGCGCTTTGA3′ and reverse 5′AGTACTAGTTTACAACCCGTGCCCTCC3′). An initial denaturation of 5 min at 94°C was followed by 30 cycles of amplification (30 s at 94°C, 30 s at 62°C and 90 s at 72°C), with a final extension of 10 min at 72°C. The products of this PCR assay were sequenced using bi-directional sequencing on an Applied Biosystems 3700 automated DNA sequencing machine in the GeneCore Biotechnologies.

### Reverse transcription PCRs (RT-PCR)

The total RNA of cells was extracted using Trizol reagent according to the manufacturer's protocol (TaKaRa, Japan). Complementary DNA (cDNA) of cells was synthesized from 1 µg of total RNA using the ImProm-II™ Reverse Transcription System (Promega, USA). PCR was performed using Pfu DNA polymerase and the following primers for HPV 18 E6 (forward: 5′AAGATTTATTTGTGGTGT 3′, reverse: 5′GCTGGATTCAACGGTTTC 3′) and for HPV 18 E7 (forward: 5′TATGCATGGACCTAAGGC 3′, reverse: 5′CAGCCATTGTTGCTTACT 3′). An initial denaturation of 5 min at 94°C was followed by 30 cycles of amplification (30 s at 94°C, 30 s at 57°C and 30 s at 72°C), with a final extension of 10 min at 72°C.

### Western blot analysis

Protein samples from the cells were extracted with the ProteoExtract Subcellular Proteome Extraction Kit (Calbiochem, USA) according to the manufacturer's instructions. The protein samples were boiled for 5 min in 1×SDS sample buffer, and 50 µg of the proteins were loaded in wells of a 15% sodium dodecyl sulfate-polyacrylamide electrophoresis (SDS-PAGE) gel. Following separation, the proteins were transferred to a nitrocellulose membrane, after blocking with 5% nonfat dry milk for 1 h at room temperature, the membrane was incubated overnight at 4°C with primary antibodies specific for HPV 18 E6, HPV 18 E7 (Santa Cruz, USA) and β-actin (Cell Signaling Technology, USA). The immunoblot analysis was performed via enhanced chemiluminescence.

### RNA interference

Synthetic target HPV 18 E7 siRNAs sequences (Table 4) were obtained from GenePharma (Shanghai, China). The siRNA was separately cloned into pGPH1 vector and this was verified through DNA sequencing analysis performed at Shanghai GenePharma Company. In brief, 5×10^5^ Hep G2 cells were cultured in 6-well plates using 2 mL of DMEM supplemented with 10% FBS. After incubation for 24 h when cells were about 50% confluent, they were transfected with 2.16 µg of the siRNA-E7 pools (HPV 18 E7-63, HPV 18 E7-112 = 1∶1) or NC-E7 (Negative control), using Lipofectamine™ 2000 (Invitrogen, USA) transfection reagent at a siRNA: Lipofectamine™ 2000 ratio of 1.08 µg: 2 µL according to the manufacturer's instructions. Transfection was terminated 4∼6 h later by replacing the medium with 2.0 mL fresh medium containing 10% FBS.

**Table 4:** siRNA targeting HPV 18 E7 sites and sequences.

Name	Sequence	
HPV 18 E7-63	Sense	5′- CGGUUGACCUUCUAUGUCATT-3′
	Anti -sense	5′-UGACAUAGAAGGUCAACCGGA-3′
HPV 18 E7-74	Sense	5′-CUAUGUCACGAGCAAUUAATT -3′
	Anti -sense	5′-UUAAUUGCUCGUGACAUAGAA -3′
HPV 18 E7-112	Sense	5′- CGAUGAAAUAGAUGGAGUUTT-3′
	Anti -sense	5′-AACUCCAUCUAUUUCAUCGTT -3
Negative Control FAM-siRNA	Sense	5′-UUCUCCGAACGUGUCACGUTT-3′
	Anti -sense	5′-ACGUGACACGUUCGGAGAATT-
Negative Control siRNA	Sense	5′- UUCUCCGAACGUGUCACGUTT -3′
	Anti -sense	5′-ACGUGACACGUUCGGAGAATT-3

### EdU staining for cell proliferation

EdU is a novel alternative for BrdU assays to directly measure active DNA synthesis or the S-phase DNA synthesis of the cell cycle. At 0 h, 12 h, 24 h, 36 h, 48 h, 60 h, and 72 h after transfection, cells transfected with siRNA-E7 or NC-E7 were incubated with 50 µM EdU for two hours at 37°C using the Click-iT® EdU Alexa Fluor® 594 Imaging Kit (Invitrogen, USA) according to the manufacturer's instructions. After fixation, cells were treated with the reagent containing Alexa 594 azide, followed by the counterstaining with Hoechst 33342 for detection and imaging using fluorescence microscopy.

### Annexin V-FITC apoptosis assays

Apoptosis of Hep G2 cells were determined using the Annexin V-FITC Apoptosis Detection Kit (Calbiochem, USA) according to the manufacturer's protocol. In brief, at 24 h, 48 h and 72 h after being transfected with siRNA-E7 or NC-E7, cells were washed twice in cold PBS and then resuspended in 1× binding buffer at a concentration of 1×10^6^ cells/mL. An aliquot of 500 µl solution (5×10^5^ cells) was then transferred to another tube containing 1.25 µL Annexin V-FITC and the cells were gently vortexed and incubated for 15 min at room temperature in the dark. Then an aliquot of 500 µL cold 1×binding buffer and 10 µL PI were added and the cells were analyzed with a FACScalibur flow cytometer within one hour.

### Expression profiling of E7-related genes in Hep G2 cells using real-time PCR human cell cycle and apoptosis arrays

The real-time PCR microarrays of cell cycle and apoptosis signaling pathways were purchased from Qiagen (CAT: PAHS-020 and PAHS-01) and were used according to the manufacturer's instructions. The siRNA E7-63 was separately cloned into pGPH1 vector and this was verified through DNA sequencing analysis performed at Shanghai GenePharma Company. In brief, the real-time PCR reactions (40 cycles) were performed with sequential incubations of 10 min at 95°C, 15 seconds at 95°C, and 1 min at 60°C. The fold- change for each gene from experimental group and control group was expressed as 2^−ΔΔCT^. If the fold change was greater than 2, then the result was reported as a fold up-regulation. If the fold change was less than 2, then the negative inverse of the result was reported as a fold down-regulation.

### In situ hybridization on tissue microarrays

Tissue samples and microarrays were obtained from National Engineering Center for BioChip at Shanghai following written informed consent according to an established protocol approved by the Ethic Committee of Second Military Medical University. Datas do not contain any information that may lead to the identification of the patients. All studies were approved by the Institutional Ethical Review Board at the Shantou University Medical College. Tissue microarrays were constructed of 2-mm cores of patient tissues taken from representative areas of Hepatocellular carcinoma. Analysis was performed on the 100 independent cases including 28 of grade I, 65 of grade II, 7 of grade III. We performed in situ hybridization (ISH) using a Subtype-specific HPV probe on all cases. The ISH screening was performed using a digoxigenin-labeled HPV probe cocktail detecting DNA-HPV types 16 and 18 (Triplex International Biosciences, Fujian, China). Sections were counterstained with hematoxylin, dehydrated, and mounted. Positive controls (HeLa cells) were served with ISH for HPV. Negative controls were prepared by substituting PBS for the primary antibody. Cases with HPV signals in the punctate nuclear or diffuse staining of tumor cells were determined to be positive.
